# Sarcopenia is independently associated with diabetic foot disease

**DOI:** 10.1038/s41598-017-08972-1

**Published:** 2017-08-21

**Authors:** Qingfeng Cheng, Jinbo Hu, Ping Yang, Xueting Cao, Xuefeng Deng, Qin Yang, Zhiping Liu, Shumin Yang, Richa Goswami, Yue Wang, Ting Luo, Kun Liao, Qifu Li

**Affiliations:** Department of Endocrinology, The First Affiliated Hospital of Chongqing Medical University, 400016 Chongqing, China

## Abstract

The aim of this study was to investigate the association of sarcopenia and diabetic foot disease (DFD) in a cross-sectional study. Body composition was assessed using dual-energy X-ray-absorptiometry (DXA) among 1105 patients with type 2 diabetes (120 patients with newly diagnosed DFD [DFD duration less than 3 months]). Severity of DFD was assessed, referring to foot ulcers, Wagner grade and the percentage of amputation. Skeletal muscle index (SMI) was calculated, and sarcopenia was defined as SMI less than 7.0 kg/m^2^ (in men) or 5.4 kg/m^2^ (in women). SMI was significantly decreased in patients with DFD compared to patients without (6.79 ± 1.20 vs. 7.21 ± 1.05 kg/m^2^, *P* < 0.001). The percentage of sarcopenia in DFD patients was more than double than those without DFD (35.3% vs. 16.4%, *P* < 0.001). Multivariable logistic regression analysis showed that sarcopenia was independently associated with DFD (OR 2.05[95% CI 1.15,3.89], *P* = 0.027) after controlling confounders including age, diabetic duration and diabetic chronic complications. In DFD group, patients with sarcopenia exhibited more foot ulcers, higher Wagner grade and greater percentage of amputation compared to patients without sarcopenia. We conclude that sarcopenia is independently associated with DFD. Worse prognosis is seen in patients with DFD accompanied by sarcopenia.

## Introduction

Diabetic foot disease (DFD) is the leading cause of non-traumatic limb amputation^1^.The risk of foot problems in patients with diabetes dramatically increased in recent decades in China^2^. DFD not only affects the quality of life of patients with diabetes, but challenges health systems around the world^3^. Although peripheral arterial disease (PAD), diabetic neuropathy and infection are considered to be the major causes of DFD, the intrinsic mechanism of DFD still needs to be elucidated^1–3^.

The prevalence of sarcopenia reaches up to 15% in patients with type 2 diabetes (T2D)^4^. Sarcopenia is associated with risks of cardiovascular events such as critical limb ischemia (CLI) and cardiovascular-specific mortality^5, 6^. Impaired function of neuromuscular system was also observed in patients and rodent models with sarcopenia^7, 8^. It was reported that oxidative stress, chronic inflammation, mitochondrial dysfunction and reduced regenerative capacity not only contribute to the pathophysiology of sarcopenia^9–12^, but also closely correlate to the incidence of DFD^13–16^. Both sarcopenia and DKD have similar underlying mechanisms, but whether sarcopenia is associated with DFD has yet been reported.

Here, we conducted a cross-sectional study to investigate the association of sarcopenia with DFD. Dual-energy X-ray absorptiometry (DXA) was used to evaluate lean tissue in 1105 patients with T2D. The severity of DFD was evaluated referring to foot ulcers, Wagner grade and the percentage of amputation.

## Results

Clinical characteristics of subjects are presented in Table 1. A total of 1105 patients with type 2 diabetes were included (120 patients with newly diagnosed DFD [duration 1.0 (0.3, 2.0) months], and 985 patients without DFD). Compared to subjects without DFD, DFD patients exhibited higher levels of white blood cell, HbA1c and creatinine, and lower levels of hemoglobin, BMI and ABI. The percentage of DR, DPN, DKD and PAD were significantly higher in patients with DFD. Anti-diabetic and anti-hypertensive medications such as metformin and ACEI/ARB are also shown in Table 1.

**Table 1:** Characteristics of patients with type 2 diabetes with or without diabetic foot disease.

	DFD (N = 120)	non-DFD (N = 985)	*P* value
Male/Female (person)	67/53	565/420	0.845
Age (year)	66.84 ± 11.18	64.35 ± 9.32	0.007
Duration of Diabetes (year)	9(5,15)	8(4,13)	0.086
History of Hypertension (%)	63.1	58.1	0.361
History of Smoking (%)	53.70	39.10	0.032
BMI (kg/m^2^)	24.03 ± 3.55	24.85 ± 3.23	0.010
Waist Circumference (cm)	94.9 ± 9.74	94.85 ± 9.12	0.953
Hemoglobin (g/L)	119.55 ± 20.68	134.09 ± 18.19	<0.001
White Blood Cell (*10^9^/L)	8.14 ± 3.81	6.34 ± 1.44	<0.001
Neutrophil (*10^9^/L)	6.11 ± 3.83	4.07 ± 1.22	<0.001
Total Cholesterol (mmol/L)	4.02 ± 1.16	4.25 ± 1.13	0.040
Triglyceride (mmol/L)	1.37 ± 0.70	1.87 ± 1.63	0.001
HDL-c (mmol/L)	1.11 ± 0.38	1.15 ± 0.37	0.334
LDL-c (mmol/L)	2.50 ± 1.02	2.58 ± 0.94	0.413
hs-CRP (mg/L)	6.92 (2.34,20.00)	0.84 (0.44,1.79)	<0.001
HbA1c (%)	9.38 ± 2.42	8.43 ± 2.23	<0.001
UACR (mg/g)	35.7 (10.35,158.95)	12.25 (4.2,50.6)	0.001
Creatinine (μmol/L)	76.37 (59.25,97.51)	71.13 (59.02,87.56)	<0.001
Uric Acid (μmol/L)	302.87 ± 114.49	312.88 ± 88.9	0.275
Ankle Brachial Index	1.03 (0.96,1.18)	1.16 (1.12,1.21)	<0.001
**Chronic Diabetic Complications**			
Diabetic Retinopathy (%)	47.9	26.7	<0.001
Diabetic Peripheral Neuropathy (%)	81.5	70.9	0.012
Diabetic Kidney Disease (%)	42.7	31.9	0.047
Coronary Heart Disease (%)	15.1	15.8	1.000
PAD (%)	24.4	1.3	<0.001
**Anti-diabetic and Anti-hypertensive Medications**			
Metformin (%)	53.1	68.9	0.002
Insulin Secretagogues (%)	15.3	33.8	<0.001
Acarbose (%)	22.9	26.9	0.341
Insulin (%)	73.3	62.7	0.049
ACEI/ARB (%)	33.1	52.9	<0.001
CCB (%)	33.8	32.8	1.000
Diuretic (%)	12.2	4.6	0.004

Data are presented with mean ± SD, median (interquartile range), or %. BMI: body mass index, HDL-c: high density lipoprotein cholesterol, LDL-c: low density lipoprotein cholesterol, hs-CRP: high-sensitivity c-reactive protein, UACR: urinary albumin-to-creatinine ratio, HbA1c: glycosylated hemoglobin, PAD: peripheral arterial disease, ACEI: angiotensin-converting enzyme inhibitors, ARB: angiotensin receptor blocker, CCB: calcium channel blocker.

**Figure 1:**
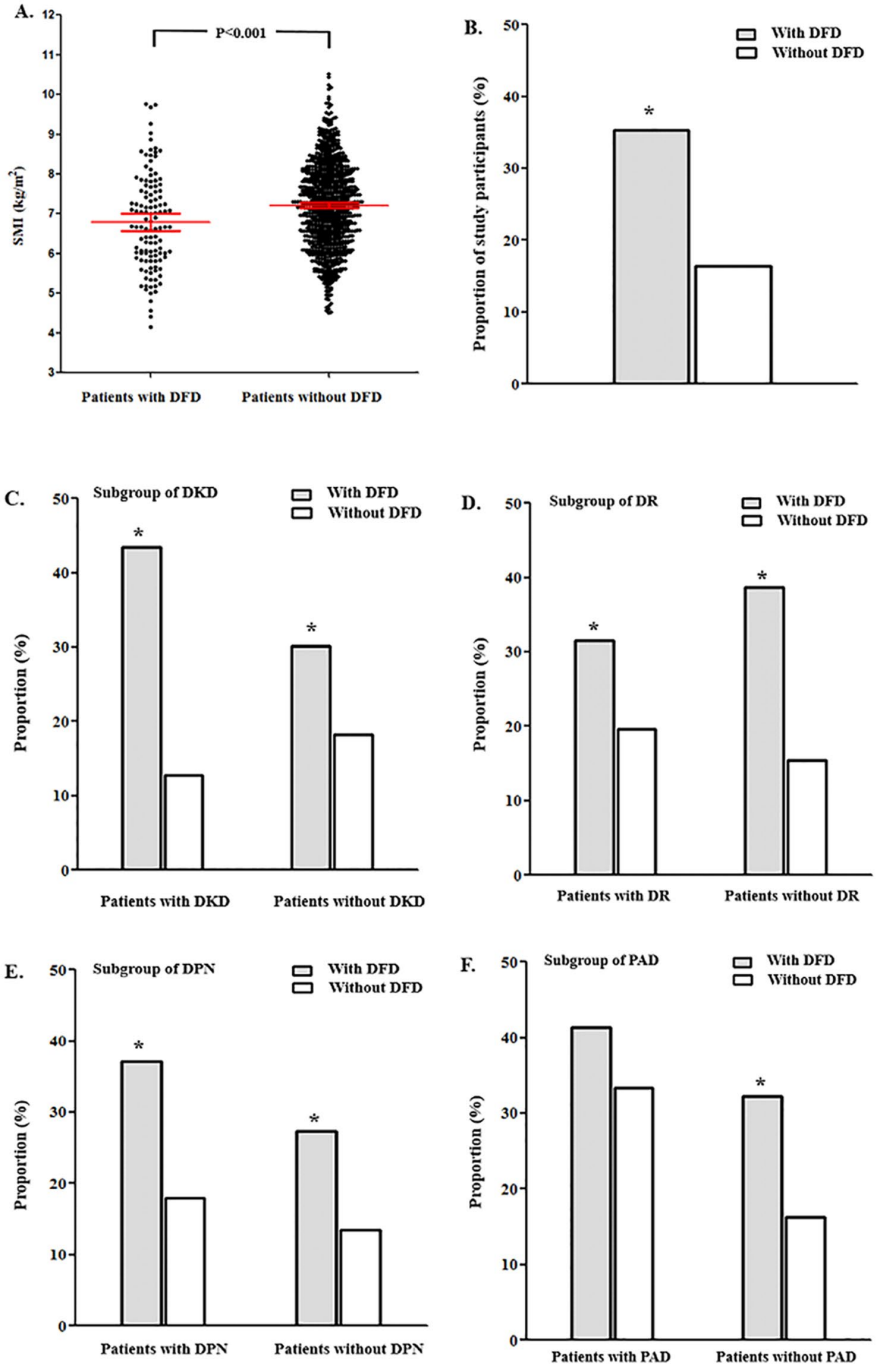
SMI and percentage of sarcopenia in patients with or without DFD. (**A**) Skeletal muscle index (SMI) in patients with or without DFD. Red lines represent mean and 95% confidence interval (CI). (**B**) The percentage of sarcopenia in patients with or without DFD. Subgroup analyses were performed based on chronic diabetic complications including DPN(**C**), PAD(**D**), DR(**E**) and DKD(**F**). *P < 0.05 compared to patients without DFD (the *P* Value based on Pearson Chi-Square test).

Two hundred and four patients are diagnosed as sarcopenia. Compared to patients without sarcopenia, patients with sarcopenia exhibited higher proportion of DPN (80.0% vs. 70.3%, *P* = 0.007) and PAD (8.1% vs. 3.1%, *P* = 0.004). The percentage of sarcopenia in DFD patients was more than double than patients without DFD (35.3% vs. 16.4%, *P* < 0.001). SMI was significantly decreased in patients with DFD (6.79 ± 1.20 vs. 7.21 ± 1.05 kg/m^2^, *P* < 0.001) (Fig. 1A). Subgroup analyses showed that in patients with chronic diabetic complications such as DPN, DR or DKD, the percentage of sarcopenia in DFD patients were also significantly higher than those without DFD; while in subgroup of PAD, the percentage of sarcopenia in DFD patients were higher than non-DFD patients without statistical significance (Fig. 1B).

DXA-measured body compositions are summarized in Supplementary Table 1s. The extremity lean mass was significantly decreased in in patients with DFD compared to patients without DFD, while the trunk lean mass was similar. Compared to patients without DPN or PAD, SMI was also significantly decreased in patients with DPN or PAD (7.11 ± 1.06 vs. 7.30 ± 1.10 kg/m^2^, *P* = 0.012 for DPN; 6.53 ± 0.94 vs. 7.17 ± 1.07 kg/m^2^, *P* < 0.001 for PAD), and the percentage of sarcopenia was significantly higher in patients with DPN or PAD (DPN: 20.5% vs. 13.3%, *P* = 0.007; PAD: 39.0% vs. 18.7%, *P* = 0.004) (Supplementary Table 1s).

Crude and multivariable-adjusted models were used for evaluating the relationship between potential risk factors and DFD. The crude model showed that a higher SMI was associated with a lower risk of DFD (OR 0.67 [95% CI 0.55,0.81], *P* < 0.001). After adjusting for potential confounders of DFD, the relationship between SMI and DFD remains the same [0.74(0.57,0.97), *P* = 0.031], and patients with sarcopenia exhibited higher risk of DFD compared to subjects without sarcopenia [2.06(1.08,3.95), *P* = 0.029] (Table 2).

**Table 2:** Univariate and multivariable analysis for logistic regression of diabetic foot disease.

Parameters	Crude^*^		Models^‡^	Multivariable^†^	
	OR(95% CI)	P Value		OR(95% CI)	*P* Value
Age (year)	1.03(1.01,1.05)	0.007	Multivariable analysis for Sarcopenia		
BMI (kg/m^2^)	0.92(0.87,0.98)	0.011	Model 1	2.48(1.63,3.77)	<0.001
HbA1c (%)	1.18(1.09,1.28)	<0.001	Model 2	2.49(1.14,4.41)	0.002
PAD	23.74 (11.71,48.13)	<0.001	Model 3	2.20(1.24,3.93)	0.007
DPN	1.81(1.12,2.94)	0.016	Model 4	2.15(1.21,3.83)	0.009
DR	2.53(1.71,3.73)	<0.001	Model 5	2.40(1.36,4.25)	0.003
DKD	2.01(1.22,2.98)	0.006	Model 6	2.12(2.18,3.81)	0.012
SMI	0.67(0.55, 0.81)	<0.001	Model 7	2.14(1.18,3.86)	0.012
Sarcopenia	2.77(1.84,4.18)	<0.001	Model 8	2.06(1.08,3.95)	0.029

*Crude: Univariate logistic regression analyses for the association between potential risk factors and DFD. ^†^Multivariable: Multivariable logistic regression analyses to test whether sarcopenia is independently associated with DFD. In the univariate or multivariable logistic regression analyses, DFD was used as the dependent.

^‡^Models were established as follows:

Model 1: Adjusted for gender, age, duration of T2D, duration of DFD;

Model 2: Model 1 + adjusted for BMI, smoking, hypertension, serum creatinine concentration;

Model 3: Model 2 + adjusted for White Blood Cell and HbA1c;

Model 4: Model 3 + adjusted for DKD;

Model 5: Model 4 + adjusted for DR;

Model 6: Model 5 + adjusted for DPN;

Model 7: Model 6 + adjusted for PAD;

Model 8: Model 7 + adjusted for medications, including metformin, insulin secretagogues, insulin, ACEI/ARB and diuretics.

Patients with DFD were grouped into presence of sarcopenia (DFD with sarcopenia) and absence of sarcopenia (DFD without sarcopenia). The duration of DFD was similar in DFD patients with or without sarcopenia (1.0 [0.3, 2.0] vs. 1.0 [0.2, 2.0], *P* = 0.721). Foot ulcers, Wagner grade and amputation in patients with DFD are shown in Table 3. Compared to DFD patients without sarcopenia, DFD patients with sarcopenia exhibited more ulcers (the percentage of 0 ~ 4 or more ulcers were 9.5%, 33.3%, 28.6%, 23.8%, 4.8% in DFD patients with sarcopenia, respectively; and 22.1%, 48.1%, 20.8%, 7.8%, 1.3% in DFD patients without sarcopenia, respectively; *P* = 0.022), a greater ulcer size (6.5[2.1,12.0] vs. 3.0[1.2,6.0] cm^2^, *P* = 0.007), and an increased Wagner grade (the percentage of Grade 1 ~ Grade 5 were 37.8%, 22.7%, 26.1%, 11.8%, 1.7% in DFD patients with sarcopenia, respectively; and 46.8%, 24.7%, 22.1%, 6.5%, 0% in DFD patients without sarcopenia, respectively; *P* = 0.003). The percentage of amputation was significantly higher in DFD patients with sarcopenia compared to DFD patients without sarcopenia (21.4% vs. 7.8%, *P* = 0.044) (Table 3).

**Table 3:** Foot ulcers, Wagner grade and amputation in DFD patients with or without sarcopenia.

	DFD with Sarcopenia (N = 43)	DFD without Sarcopenia (N = 77)	*P* Value^†^
**Duration of DFD**	1.0 (0.3, 2.0)	1.0 (0.2, 2.0)	0.721
**Foot ulcers**			
Number of ulcers			
No ulcer (%)	9.5	22.1	0.022
1 ulcer (%)	33.3	48.1	
2 ulcers (%)	28.6	20.8	
3 ulcers (%)	23.8	7.8	
4 or more ulcers (%)	4.8	1.3	
Ulcer size (cm^2^)	6.5 (2.1,12.0)	3.0 (1.2,6.0)	0.007
Wagner grade*			
Grade 1 (%)	37.8	46.8	0.003
Grade 2 (%)	22.7	24.7	
Grade 3 (%)	26.1	22.1	
Grade 4 (%)	11.8	6.5	
Grade 5 (%)	1.7	0	
**Amputation (%)**	21.4	7.8	0.044

*The Wagner system assesses ulcer depth and the presence of osteomyelitis or gangrene by using the following grades: grade 1 (partial/full thickness ulcer), grade 2 (probing to tendon or capsule), grade 3 (deep with osteitis), grade 4 (partial foot gangrene), and grade 5 (whole foot gangrene). ^†^For ulcer size, the *P* Value based on non-parametric test; for amputation, the *P* Value based on Pearson Chi-Square; for number of ulcers and Wagner grade, the *P* Value based on Fisher’s exact test.

## Discussion

Our study indicates that sarcopenia is associated with DFD, and worse prognosis is seen in patients with DFD accompanied by sarcopenia. Of note, the relationship between sarcopenia and DFD was independent of the known risk factors such as gender, age, hypertension, glycemia and chronic diabetic complications.

Neuropathy and vascular disease are known DFD risk factors. Previous studies have revealed that neuropathy and vascular disease are associated with sarcopenia. A cross-sectional study indicated that old adults with sarcopenia are more prone to lose more motoneuron than those without sarcopenia^8^. Aged mice with sarcopenia exhibited the accumulation of proteins such as vimentin and tau5 and the ultrastructure that electron-dense aggregates within axons in peripheral nerves, suggesting impaired mechanisms for axonal transport and protein turnover^17^. A retrospective study which recruited 64 patients with critical limb ischemia (CLI), including 28 patients with sarcopenia and 36 without, showed that 5-year survival rate was about three times lower in CLI patients with sarcopenia^5^. Another cross-sectional study reported that sarcopenia is associated with lower skeletal muscle capillarization in older adults^18^. The present cross-sectional study found that patients with sarcopenia exhibited higher proportion of neuropathy and PAD, which are consistent with previous studies. Therefore, neuropathy and vascular lesions might associate sarcopenia with DFD.

It should be noted that apart from known DFD risk factors^19^, sarcopenia is independently associated with DFD. In the subgroup analyses of patients with DPN, DR or DKD, the percentage of sarcopenia in DFD patients was significantly higher than patients without DFD. However, in the subgroup analysis of PAD, the percentage of sarcopenia in DFD patients was not significantly higher than patients without DFD, which might be attributed to the limited sample size of PAD subgroup. Furthermore, we established eight multivariable logistic regression models to test the independent relationship between sarcopenia and DFD. Potential confounders were adjusted stepwise, including gender, age, duration of T2D, duration of DFD, BMI, smoking, hypertension, serum creatinine concentration, levels of white blood cell, HbA1c, DKD, DR, DPN, PAD, metformin, insulin secretagogues, insulin, ACEI/ARB and diuretics. Both in the crude model and multivariable models, sarcopenia was independently correlated to DFD, suggesting sarcopenia as a risk factor for DFD.

The molecular mechanism of sarcopenia correlating to DFD has not been intensively explored. Based on previous reports, several possible mechanism might explain this association. Firstly, skeletal muscle has been considered to be an endocrine organ, myokines and myometabolites secreted by skeletal muscle mediate communications between muscle and other organs^20^. Patients with sarcopenia has changed production of myokines for muscle^20^, which might connecting sarcopenia with DFD. Secondly, muscle weakness has been associated with higher risk of foot injury^21^, which is a common cause for DFD. Thirdly, both sarcopenia and DFD have similar underlying mechanisms including oxidative stress, chronic inflammation, mitochondrial dysfunction^13–16^. Muscle overproduction of reactive oxygen and nitrogen species are observed in sarcopenia, and the risk of sarcopenia is greatly reduced by muscle-specific inhibition of oxidative stress^9, 22^. Observational studies and biopsy studies strengthen the association between chronic low-grade inflammatory profile and sarcopenia^10^. Mitochondrial dysfunction in skeletal muscle has been associated with the pathogenesis of sarcopenia, and improved quality control of mitochondria is thought to be a potential intervention for managing sarcopenia^11^. Furthermore, there is reduced regenerative capacity of skeletal muscle in sarcopenia, and decline in regeneration of stem cells is also well recognized in sarcopenia^11, 12^. Muscle overproduction of reactive oxygen and nitrogen species in sarcopenia might mediate the progression of neuropathy and vascular lesions and associate sarcopenia with DFD. The main strength of our study is a relatively large sample size with DXA-based body composition measurement. As sarcopenia is related to DFD, and patients with DFD accompanied by sarcopenia show worse prognosis, attention for sarcopenia and interventions to prevent sarcopenia might be important. However, several caveats in our study merit discussion. The walking speed on a 4-meter course was not assessed. Considering long-term inactive skeletal muscle could lead to sarcopenia, we excluded patients whose DFD duration more than 3 months. Nevertheless, the duration of DFD on the incidence of sarcopenia could not be completely ruled out.

There are some limitations of the current study. This study aimed at investigating the association of sarcopenia and DFD, therefore, subjects with type 2 diabetes (with and without DFD) were recruited. Due to a high risk of sarcopenia in patients with type 2 diabetes, the detection signal bias might be caused. In addition, subjects were selected among hospitalized patients in a single center, which might lead to an admission rate bias. When looking at the effect size of sarcopenia on DFD, these bias should be considered. As a cross-sectional study, our objective was to investigate whether sarcopenia was independently associated with DFD. We could not conclude whether sarcopenia is a cause of DFD or a result of it, and prospective or interventional studies in the future are needed to unravel this question. As sarcopenia was not noticed in clinic yet, our study might provide a reminder for paying attention to sarcopenia and its effects on DFD.

In conclusion, it is shown that sarcopenia is independently associated with DFD, and worse prognosis is seen in patients with DFD accompanied by sarcopenia. Our study expands the current knowledge on the relationship between sarcopenia and DFD, and highlights sarcopenia might be an important risk factor for DFD in T2D patients.

## Methods

### Study design and Participants

This study was performed at the First Affiliated Hospital of Chongqing Medical University, China, from June 2013 to December 2015. T2D was diagnosed based on a standard oral glucose tolerance test (OGTT) or previous medical records. Inclusion criteria: patients with T2D. Exclusion criteria: duration of DFD > 3 months; individuals with age < 20 or > 85 years; severe heart failure (New York Heart Association Class II–IV); severe liver impairment (liver enzyme ALT ≥ 3-fold the upper limit of normal range); severe renal dysfunction (estimated glomerular filtration rate [eGFR] < 30 ml/min/1.73 m^2^); a history of thyroid or adrenal diseases; a history of malignant tumor.

Of the 1539 patients with T2D who were interviewed, 1105 T2D patients with available data were recruited. Informed consent was obtained from all participants. Ethical Committee of the First Affiliated Hospital of Chongqing Medical University approved this study, and all methods were performed in accordance with our local guidelines and clinical regulations.

### Clinical Procedures and Laboratory Measurements

Experienced physicians collected and recorded medical and social history including alcohol and tobacco use. All subjects underwent physical anthropometry measurements including height, weight, waist circumference (WC), hip circumference (HC), systolic blood pressure (SBP), and diastolic blood pressure (DBP). Body mass index (BMI) was calculated by dividing weight by the square of height. A biochemical analyzer was used to measure plasma glucose levels (BS-380; Mindray Medical International, Shenzhen, China). Glycosylated hemoglobin (HbA1c) was measured using boronate affinity high performance liquid chromatography (Trinity Biotech, *ultra* ^2^, Trinity Biotech, Dublin, Ireland). Serum lipids including total cholesterol (TC), triglycerides (TG), high-density lipoprotein cholesterol (HDL-c), and low-density lipoprotein cholesterol (LDL-c) were measured enzymatically by an automatic analyzer (Model 7080; Hitachi, Tokyo, Japan) with reagents purchased from Leadman Biochemistry Co. Ltd. (Beijing, China). Serum creatinine, urinary creatinine and albumin were measured with an automatic biochemical analyzer (Modular DDP, Roche). Urinary micro-albuminuria to creatinine ratio (UACR) was calculated.

### Chronic Diabetic Complications Assessment

Evaluation for chronic diabetic complications included DFD, diabetic peripheral neuropathy (DPN), peripheral artery disease (PAD), diabetic retinopathy (DR) and diabetic kidney disease (DKD). DFD was diagnosed according to the National Institute for Health and Care Excellence guidelines^1, 19^. To be specifically, DFD was defined as active diabetic foot problem: ulceration, spreading infection, critical ischaemia, gangrene, suspicion of an acute Charcot arthropathy, or an unexplained hot, red, swollen foot with or without pain. All patients with DFD were assessed by a trained diabetic care nurse; size and number of foot ulcer were assessed, and history of lower-limb amputation was recorded; the Wagner grade system was used to evaluate ulcer depth and the presence of osteomyelitis or gangrene: grade 1 (partial/full thickness ulcer), grade 2 (probing to tendon or capsule), grade 3 (deep with osteitis), grade 4 (partial foot gangrene), and grade 5 (whole foot gangrene)^23^. DPN was evaluated by neuropathy manifestation and electromyography. PAD was defined as an ankle-brachial pressure index (ABI) less than 0.9 with further confirmed by ultrasonography or angiography. Other chronic diabetic complications such as DR and DKD were evaluated according to the guidelines of American Diabetes Association (ADA) 2012^19^.

### Body Composition and Sarcopenia Assessment

Body composition was measured using DXA Hologic scanner (Hologic Discovery QDR® Series, Bedford, MA, USA) by a trained technician. All standard procedures were performed according to previous studies^24^. Hologic Whole Body DXA reference database software was used to estimate the regional and whole body lean tissue. Skeletal muscle index (SMI) was calculated as appendicular skeletal muscle mass divided by body height in meters squared. Sarcopenia was defined as SMI less than 7.0 kg/m^2^ (in men) or 5.4 kg/m^2^ (in women)^25, 26^.

### Statistical Analyses

The assumption of normality and homogeneity of variances were tested by one sample Kolmogorov-Smirnov tests. For normally distributed variables, data were presented as mean ± SD and tested by t-test; for non-normally distributed variables, data were presented as the medians (interquartile range) and tested by nonparametric two-independent samples Kolmogorov-Smirnov tests. Categorical variables were reported as frequencies and proportion, and Pearson Chi-square tests or Fisher’s exact tests were used for group comparisons.

Univariate and multiple stepwise logistic regression were conducted to test the associations between sarcopenia with DFD. In the multiple stepwise logistic regression, crude model showed that age, HbA1c, PAD, DPN, DR, DKD and sarcopenia were risk factors for DFD. To examine whether sarcopenia was independently associated with DFD, eight models were established for the multivariable logistic regression analyses: model 1 adjusted for gender, age, duration of T2D, duration of DFD; model 2 further adjusted for BMI, smoking, hypertension, serum creatinine concentration; model 3 further adjusted for levels of white blood cell and HbA1c; model 4 further adjusted for DKD; model 5 further adjusted for DR; model 6 further adjusted for DPN; model 7 further adjusted for PAD; model 8 further adjusted for medications, including metformin, insulin secretagogues, insulin, ACEI/ARB and diuretics. Statistical analyses were performed using SPSS software (version 13.0), *P* values of <0.05 was considered statistically significant.

## Electronic supplementary material

Supplementary Information
