# Supplementary material for: Sarcopenia is independently associated with diabetic foot disease
Source: Sci Rep. 2017 Aug 21;7:8372. doi: 10.1038/s41598-017-08972-1 (PMC5566791; doi:10.1038/s41598-017-08972-1)
Supplement: Supplementary file 1 — Supplementary Information [file 41598_2017_8972_MOESM1_ESM.docx]

Sarcopenia is independently associated with diabetic foot disease

Qingfeng Cheng*, Jinbo Hu*, Ping Yang, Xueting Cao, Xuefeng Deng, Qin Yang, Zhiping Liu, Shumin Yang, Richa Goswami, Yue Wang, Ting Luo, Kun Liao, Qifu Li

Department of Endocrinology, The First Affiliated Hospital of Chongqing Medical University, 400016 Chongqing, China

Corresponding author: Qifu Li, MD (e-mail:[liqifu@yeah.net](mailto:liqifu@yeah.net))

^*^The two authors contributed equally to this work.

Supplement

Table 1s. DXA-measured body composition in type 2 diabetic patients.

|  | DPN | non-DPN | *P* value |  | PAD | non-PAD | *P* value |  | DFD | non-DFD | *P* value |
| --- | --- | --- | --- | --- | --- | --- | --- | --- | --- | --- | --- |
|  | (N＝740) | (N＝286) |  |  | (N＝974) | (N＝41) |  |  | (N＝120) | (N＝985) |  |
| Total Lean Mass (kg) | 45.3±8.3 | 45.86±8.73 | 0.336 |  | 41.62±6.48 | 45.48±8.43 | 0.004 |  | 44.03±8.47 | 45.62±8.42 | 0.042 |
| Head Lean (kg) | 3.59±0.41 | 3.63±0.38 | 0.233 |  | 3.45±0.37 | 3.61±0.4 | 0.013 |  | 3.52±0.38 | 3.61±0.40 | 0.048 |
| Trunk Lean (kg) | 23.04±4.09 | 23.07±4.27 | 0.909 |  | 21.61±3.39 | 23.05±4.15 | 0.029 |  | 22.95±4.20 | 23.07±4.14 | 0.591 |
| Left Arm Lean (kg) | 2.34±0.55 | 2.39±0.6 | 0.203 |  | 2.11±0.41 | 2.36±0.57 | 0.006 |  | 2.22±0.54 | 2.37±0.57 | 0.007 |
| Right Arm Lean (kg) | 2.52±0.59 | 2.57±0.65 | 0.283 |  | 2.28±0.44 | 2.54±0.61 | 0.006 |  | 2.39±0.58 | 2.56±0.61 | 0.004 |
| Left Leg Lean (kg) | 6.85±1.47 | 7.05±1.57 | 0.058 |  | 6.12±1.15 | 6.91±1.5 | 0.001 |  | 6.45±1.53 | 6.95±1.50 | 0.001 |
| Right Leg Lean (kg) | 6.95±1.50 | 7.16±1.57 | 0.055 |  | 6.06±1.37 | 7.02±1.51 | <0.001 |  | 6.59±1.63 | 7.06±1.50 | 0.002 |
| Lean Mass Height^2^ | 17.32±2.08 | 17.53±2.14 | 0.154 |  | 16.45±1.87 | 17.37±2.11 | 0.006 |  | 17.04±2.32 | 17.43±2.08 | 0.043 |
| SMI (kg/m^2^) | 7.11±1.06 | 7.30±1.10 | 0.012 |  | 6.53±0.94 | 7.17±1.07 | <0.001 |  | 6.79±1.20 | 7.21±1.05 | <0.001 |
| Sarcopenia (%) | 20.5 | 13.3 | 0.007 |  | 39.0 | 18.7 | 0.004 |  | 35.3 | 16.4 | <0.001 |

SMI: Skeletal muscle mass index, which is defined as appendicular skeletal muscle mass (ASM) divided by body height in meters squared. Sarcopenia is defined as SMI less than 7.0 kg/m^2^ and 5.7 kg/m^2^ in men and women, respectively.
